# The impact of hospitalization on mortality in patients with connective tissue disease-associated interstitial lung disease: a medical records review study

**DOI:** 10.1186/s42358-023-00343-x

**Published:** 2024-01-02

**Authors:** Anna Korogodina, Navneet Kaur, Xianhong Xie, Adhya Mehta, Krystal L. Cleven, Bibi Ayesha, Anand Kumthekar

**Affiliations:** 1 Department of Medicine, Montefiore Medical Center-Wakefield/Albert Einstein College of Medicine, Bronx, NY USA; 2 Touro University Medical Group, Stockton, CA USA; 3 Department of Epidemiology & Population Health, Albert Einstein College of Medicine, Bronx, NY USA; 4 Department of Internal Medicine, Jacobi Medical Center, Albert Einstein College of Medicine, Bronx, NY USA; 5 Division of Pulmonary Medicine, Montefiore Medical Center/Albert Einstein College of Medicine, Bronx, NY USA; 6 Division of Rheumatology, Montefiore Medical Center/Albert Einstein College of Medicine, Bronx, NY USA

**Keywords:** Interstitial lung Disease, Connective tissue Diseases, Hospitalization, Mortality

## Abstract

**Background:**

Interstitial lung disease (ILD) remains one of the most important causes of morbidity and mortality in patients with Connective Tissue Diseases (CTD). This study evaluated the impact of hospitalization on mortality in an ethnically and racially diverse cohort of CTD-ILD patients.

**Methods:**

We conducted a medical records review study at Montefiore Medical Center, Bronx, NY. We included 96 patients and collected data on demographic characteristics, reasons for hospitalization, length of stay, immunosuppressant therapy use, and mortality. We stratified our patients into two cohorts: hospitalized and non-hospitalized. The hospitalized cohort was further subdivided into cardiopulmonary and non-cardiopulmonary admissions. Two-sample tests or Wilcoxon’s rank sum tests for continuous variables and Chi-square or Fisher’s exact tests for categorical variables were used for analyses as deemed appropriate.

**Results:**

We identified 213 patients with CTD-ILD. Out of them, 96 patients met the study’s inclusion criteria. The majority of patients were females (79%), and self-identified as Hispanic (54%) and Black (40%). The most common CTDs were rheumatoid arthritis (RA) (29%), inflammatory myositis (22%), and systemic sclerosis (15%). The majority (76%) of patients required at least one hospitalization. In the non-hospitalized group, no deaths were observed, however we noted significant increase of mortality risk in hospitalized group (*p* = 0.02). We also observed that prolonged hospital stay (> 7 days) as well as older age and male sex were associated with increased mortality.

**Conclusions:**

Prolonged (> 7 days) hospital stay and hospitalization for cardiopulmonary causes, as well as older age and male sex were associated with an increased mortality risk in our cohort of CTD-ILD patients.

## Introduction

Connective tissue diseases are a group of autoimmune disorders, which include systemic sclerosis (SSc), polymyositis/dermatomyositis (PM/DM), rheumatoid arthritis (RA), Sjogren’s syndrome (SS), vasculitides, mixed connective tissue disease (MCTD), and systemic lupus erythematosus (SLE). Interstitial lung disease (ILD) is one of the most common pulmonary manifestations associated with CTD resulting in significant morbidity and mortality [1]. Connective tissue-related interstitial lung disease (CTD-ILD) is an umbrella term used to describe the inflammation and fibrosis of the lung parenchyma associated with various autoimmune disorders. The prevalence of ILD varies based on the type of CTD and the criteria used to diagnose ILD. For example, the prevalence of SSc-ILD is estimated as 1.7–4.2 per 100,000 people, and the prevalence of RA-ILD is ranging from 3.2 to 6 cases per 100,000 people [2]. As CTD-ILD is associated with high morbidity and mortality, all patients with CTD should be evaluated for ILD at presentation and periodically thereafter [1, 3].

CTD-ILD is a progressive disease, and routine clinical, radiological, and functional monitoring is crucial for identifying patients who are at a higher risk of progression. There are numerous predictors of disease progression and mortality in CTD-ILD, such as older age, male sex, steroid-refractory ILD, and usual interstitial pneumonia patterns on imaging etc [3]. Hospitalization is another risk factor for mortality associated with ILD. Patients with CTD-ILD are hospitalized for multiple causes, such as primary ILD exacerbation, acute respiratory failure secondary to pneumonia, cardiac etiologies and for management of co-morbidities. A nationwide inpatient sample database from 2006 to 2016 showed that the all-cause hospital admission rate of patients with ILD declined, but their overall mortality remained unchanged from December to April, when the highest number of hospitalizations and mortality was reported [4]. In a cohort of SSc patients who required hospitalization, 24% had a secondary diagnosis of ILD [5]. The 5-year mortality in patients with RA and ILD was 39%, and 72% of the patients had an inpatient admission over the same duration [6]. In addition to hospitalization, the reason for admission can also be helpful in identifying high-risk patients. A 10-year study examining the national inpatient sample for acute coronary syndrome showed that patients with SSc have increased inpatient mortality compared to those without SSc. Thus, it is important to identify the predictors of mortality in patients with CTD-ILD requiring inpatient hospitalization, which can help in early intervention, therapy modification, and prognostication.

Our study aims to assess the impact of hospitalization on clinical outcomes in a diverse cohort of CTD-ILD patients at a tertiary medical center.

## Materials and methods

### Study design and participants

We conducted a medical records review study at the Montefiore Medical Center. Our population of interest included adult patients ≥ 18 years old followed at Montefiore Medical Center between January 2007 and December 2018 who had a rheumatologist-diagnosed CTD and a diagnosis of ILD based on the following criteria: (a) biopsy-proven diagnosis of ILD and/or (b) two CT scans of the chest consistent with ILD (c) and/or two sets of pulmonary function tests (PFT) at least 6 months apart consistent with ILD.

### Definitions

ILD was defined as a group of conditions characterized by inflammation and progressive fibrosis, either biopsy-proven and/or with typical radiologic features, with corresponding International Classification of Diseases (ICD) 9/10 codes J84, J84.1, and J84.11. CTD diagnoses were captured through a chart review, and included ICD 9/10 codes M06.9, M32.9, M34, M35.0, M35.1, M45A, G72.4, I77.82. CTD-associated ILD was defined as the presence of ILD along with a rheumatologist-diagnosed CTD. Hospitalization status was captured through a retrospective chart review and was defined as admission to the inpatient unit. Cardiopulmonary hospitalization was defined as a hospital admission for a chief concern or admitting/primary diagnosis related to cardiopulmonary causes, including infections of the lower respiratory tract. Non-cardiopulmonary hospitalization was defined as any hospital admission unrelated to a cardiopulmonary admission/primary diagnosis. Survival was considered a primary outcome, and secondary outcomes included hospitalization type-specific survival, and survival stratified by the length of inpatient stay.

### Data collection

We queried the clinical looking glass (CLG) software to identify patients with ICD 9/10 codes corresponding to ILD (J84, J84.1, and J84.11) and two or more rheumatology visits. We collected demographic information, co-morbidities, medication history, physical examination findings, laboratory parameters, imaging parameters such as chest X-ray and computed tomography of the chest, and functional data such as pulmonary function tests.

### Statistical analysis

**Fig. 1:**
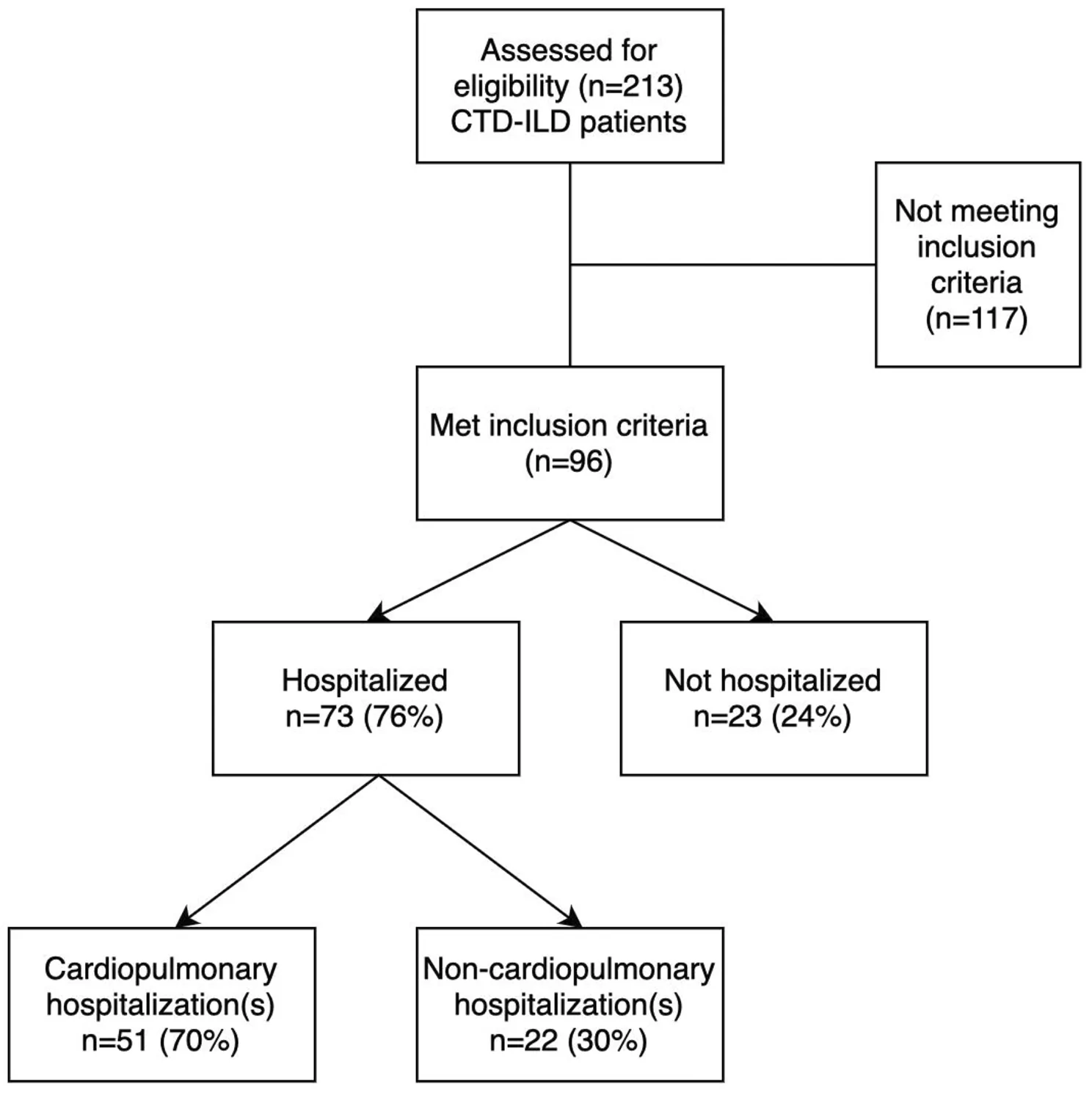
Flowchart illustrating the screening and categorization process of CTD-ILD patients based on eligibility, inclusion criteria, and hospitalization reasons

We stratified our patients into two cohorts: non-hospitalized and hospitalized. The latter cohort was further subdivided into patients with cardiopulmonary and non-cardiopulmonary hospitalizations (Fig. 1).

We analyzed the data using two-sample t-tests or Wilcoxon’s rank sum tests for continuous variables and Chi-square or Fisher’s exact tests for categorical variables, as appropriate. We used the Kaplan-Meier method for survival analysis and calculating the survival curves. The time of onset in survival analysis was considered the time of initial ILD diagnosis. To compare differences between groups we used Log-rank tests. Cox regression models were used to obtain hazard ratios with confidence intervals. All tests were two-sided, *p* < 0.05 was considered statistically significant. SAS software version 9.4 was used for the statistical analyses.

## Results

A total of 213 patients were initially identified from the CLG search between January 2007 and December 2018. Out of them, 96 patients met our study’s inclusion criteria, 73 patients (76%) had at least one hospitalization and 23 (24%) had no hospital admissions (Fig. 1).

Baseline characteristics are shown in Table 1 and were similar between both groups. In our cohort, 38 (40%) patients identified themselves as Black, 44 (54%) as Hispanics and the majority of patients were females 76 (79%). Active or former smoking history was reported by 43% of the patients. Although more smokers were identified in the hospitalized group compared with the non-hospitalized group (47% vs. 30%), the difference was not statistically significant (*p* = 0.17). Moderately severe to severe Forced Vital Capacity (FVC) predicted was 41% and 9% respectively in the hospitalized group vs. 58% and 5% in the non-hospitalized group. Diffusing capacity of the lungs of carbon monoxide (DLCO) was moderately to severely reduced in both groups, however, the difference was not statistically significant. There was a higher rate of referrals to the transplant program in the hospitalized group 17 (24%) and no referrals were made in the non-hospitalized group (*p* < 0.01). A majority of patients 78 (81%) with CTD-ILD were treated with some form of immunosuppressive therapy but was no difference in the hospitalized and non-hospitalized group.

**Table 1:** Comparative baseline characteristics of the entire cohort, hospitalized, and non-hospitalized CTD-ILD patients. Characteristics include demographics, lung function, radiographic patterns, and clinical interventions

Characteristic	Entire Cohort (n = 96)	Patients with CTD-ILD and hospitalization (n = 73)	Patients with CTD-ILD and no hospitalization (n = 23)	*P*-value
Age, mean ± SD	54.15 ± 13.97	54.82 ± 13.77	52.00 ± 14.71	0.40
Female, n (%)	76 (79)	56 (77)	20 (87)	0.39
Race, n (%)				0.10
Black	38 (40)	31 (42)	7 (30)	
Ethnicity, n (%) (Hispanic)	44 (54)	35 (53)	9 (56)	0.82
Former or active smoke, n (%)	41 (43)	34 (47)	7 (30)	0.17
Pulmonary hypertension, n (%)	28 (31)	24 (33)	4 (22)	0.36
FVC predicted, n (%)				0.37
Mild (> 76.9%)	18 (22)	17 (27)	1 (5)	
Moderate (64.3–76.8%)	21 (25)	15 (23)	6 (32)	
Severe (< 64.2%)	44 (53)	32 (50)	12 (63)	
DLCo predicted, n (%)				
Normal	11 (13)	8 (13)	3 (15)	
Mild	20 (24)	14 (23)	6 (30)	0.30
Moderate	26 (32)	23 (37)	3 (15)	
Severe	25 (30)	17 (27)	8 (40)	
UIP ILD pattern, n (%) (2 missing)	28 (30)	23 (32)	5 (23)	0.41
NSIP ILD pattern, n (%) (2 missing)	35 (37)	26 (36)	9 (41)	
Immunosuppression, (n) %	78 (81)	59 (81)	19 (83)	1.00
Transplant referral, (n) %	17 (18)	17 (24)	0 (0)	**0.01**

The median time from diagnosis of ILD to the first hospitalization was 1.42 years. The most common connective tissue disease was rheumatoid arthritis 28 (29%), followed by inflammatory myopathy 21 (22%), and systemic sclerosis 14 (15%) (Table 2).

**Table 2:** Distribution of CTD subtypes among the entire cohort, comparing patients with and without hospitalization. The table showcases the prevalence of each CTD subtype within the cohort and provides associated *P*-values

CTD subtype, n (%)	Entire Cohort (n = 96)	Patients with CTD associated ILD and hospitalization (n = 73)	Patients with CTD associated ILD and no hospitalization (n = 23)	*P*-value
SLE	9 (9)	6 (8)	3 (13)	0.84
Systemic Sclerosis	14 (15)	11 (15)	3 (13)	
Idiopathic Inflammatory Myositis	21 (22)	16 (22)	5 (22)	
Sjogren Syndrome	4 (4)	4 (5)	0 (0)	
MCTD	13 (13)	10 (14)	3 (13)	
RA	28 (29)	22 (30)	6 (26)	
Overlap Syndrome	4 (4)	2 (3)	2 (9)	
Others (Spondyloarthropathies, ANCA vasculitis)	3 (3)	2 (3)	1 (4)	

**Fig. 2:**
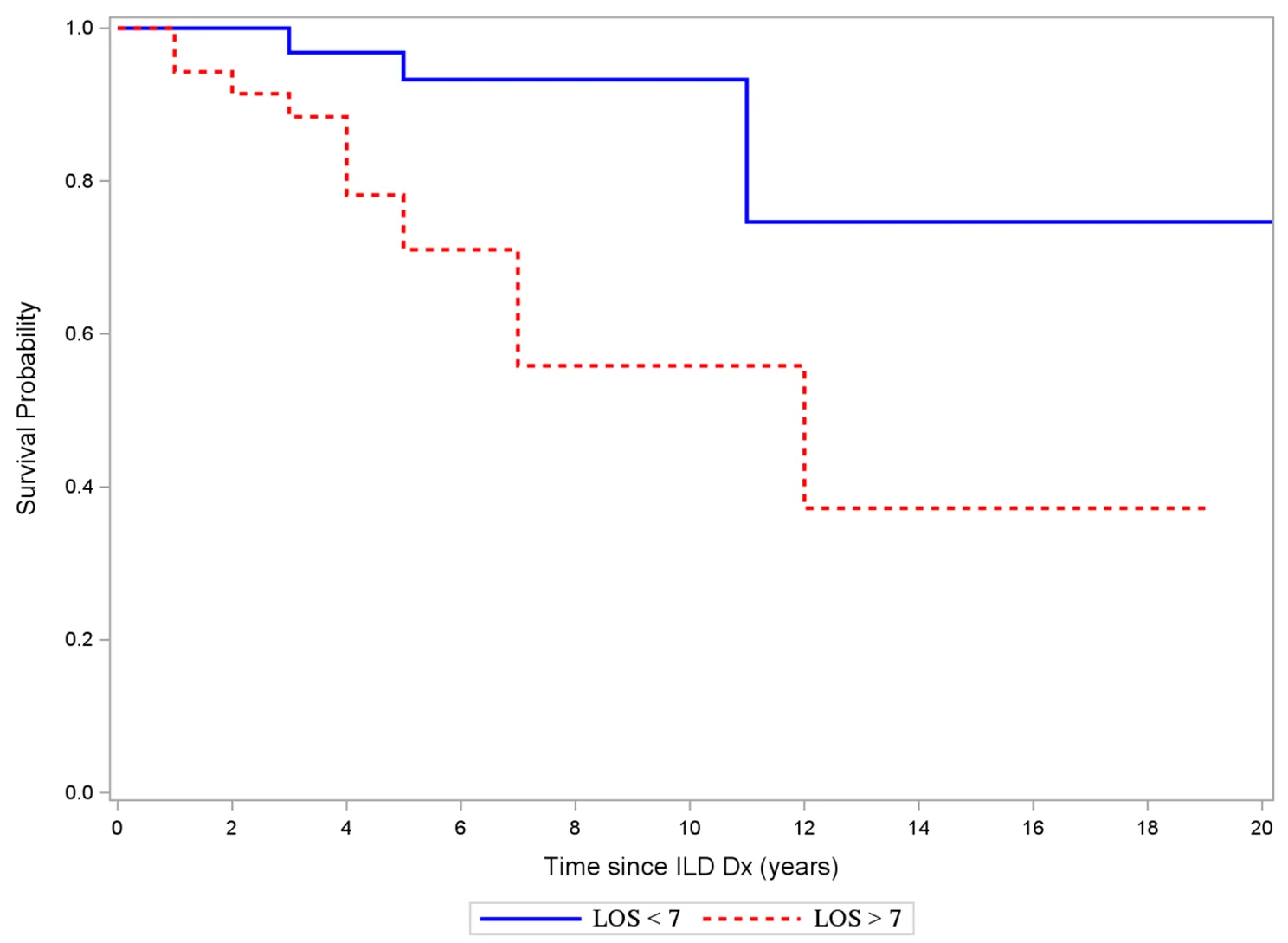
Kaplan-Meier survival curves illustrating the time from ILD diagnosis to death, differentiated by length of stay (LOS) for cardiopulmonary reasons. The solid blue line represents patients with a LOS of less than 7 days, while the dashed red line denotes those with a LOS greater than 7 days. Statistical significance between the two groups is assessed using the Log-rank test with a *p*-value of 0.01

Mortality was compared between both groups and it was significantly higher in the hospitalized group. Survival analysis showed a 5-year survival was 84% vs. 100% and a 10-year survival was vs. 76% vs. 100% for hospitalized and non-hospitalized patients, respectively (Fig. 2). No deaths were observed in the non-hospitalized group.

The hospitalized group was stratified by the reason for hospitalization: cardiopulmonary vs. non-cardiopulmonary. The majority of patients 51 (70%) were admitted for a cardiopulmonary cause (Fig. 1). Patients in the cardiopulmonary group were significantly older (Mean + SD) (57.2 + 13.1 years) than those admitted for a non-cardiopulmonary cause (Mean + SD) (49.2 + 14.8 years). Notably, cardiopulmonary hospitalization of 1 week or greater was associated with a higher risk of mortality (Table 3, HR 4.82, 95% CI: 1.37–16.92, *p* = 0.01).

**Table 3:** Bivariate hazard ratios assessing the impact of hospitalization reasons and duration on time from ILD diagnosis to death

Variable	Hazard Ratio	95% CI	*P*-value
Hospitalized for cardiopulmonary cause	3.52	0.80–15.50	0.10
LOS for cardiopulmonary cause > 7d	4.82	1.37–16.92	**0.01**
LOS for non-cardiopulmonary cause > 7d	2.90	0.97–8.69	0.06

Additionally, older age in the cardiopulmonary admissions group was also associated with increased mortality risk (Table 4, HR 1.95, 95% CI: 1.11–3.42, *p* = 0.02).

**Table 4:** Bivariate hazard ratios from Cox regression analysis evaluating various factors’ influence on time from ILD diagnosis to death

Variable	Hazard Ratio	95% CI	*P*-value
Age (per SD)	1.95	1.11–3.42	**0.02**
Female	0.18	0.07–0.50	**0.001**
Hispanic ethnicity	2.21	0.69–7.10	0.18
Former or active smoker	1.37	0.51–3.65	0.53
Pulmonary hypertension	2.32	0.86–6.25	0.10
FVC predicted, n (%)			
Mild(> 76.9%)	1 (ref)		
Moderate(64.3–76.8%)	0.55	0.09–3.29	0.51
Severe(< 64.2%)	1.13	0.29–4.37	0.86
DLCo, predicted, n (%)			
Normal/Mild	1 (ref)		
Moderate	0.78	0.13–4.69	0.79
Severe	2.45	0.59–10.27	0.22
NSIP ILD pattern	0.47	0.13–1.66	0.24
UIP ILD pattern	1.68	0.63–4.52	0.30
Immunosuppression	1.08	0.31–3.78	0.91
Transplant referral	2.12	0.67–6.70	0.20
CTD Subtype			
RA^*^	2.05	0.76–5.52	0.16
Systemic Sclerosis	1.20	0.34–4.24	0.77
Idiopathic Inflammatory Myositis	1.08	0.31–3.84	0.90
Others (Spondylarthritis, ANCA vasculitis)	4.22	0.94–18.96	0.06
SLE	0.58	0.08–4.43	0.60

*RA when compared with other CTD subtypes. Same applicable for the other CTD subtypes

## Discussion

The number or frequency of hospitalizations has been used to estimate and/or prognosticate patients’ outcomes in several chronic diseases, such as CHF or COPD [7, 8]. In many instances, recurrent hospitalizations serve as an independent risk factor for poor outcomes, and specific interventions are targeted to support vulnerable patients in the ambulatory setting and decrease readmission rates [9]. In our study spanning over 11 years in an ethnically and racially diverse community, we identified that hospitalizations for cardiopulmonary causes especially for 1 week or more and older age in patients with CTD-ILD were associated with increased mortality.

The racial and ethnic characteristics of our population of interest were remarkably different from other studies. The majority of our patients self-identified as Hispanic (54%) and Black (40%), whereas similar studies done by Brown et al. and Oldham et al. have studied primarily the impact of hospitalization in Caucasian patients with CTD-ILD, IPF, chronic hypersensitivity pneumonitis and idiopathic interstitial pneumonia [10, 11]. In the study done by Ratwani et al. [12] information about racial and ethnical characteristics of studied cohort was not readily available. This racial and ethnical distribution in our population of interest is explained by baseline demographic characteristics of the borough of the Bronx based on the 2020 Census [13]. Despite different demographic characteristics, our study demonstrated increased mortality in a hospitalized group, similar to Brown et al. [10], where both all-cause and respiratory-related hospitalizations were strongly associated with increased mortality in patients with IPF.

In patients with SSc-associated ILD, pulmonary hypertension (PH) is a leading cause of morbidity and mortality, and about 80% of patients with scleroderma will develop ILD in their lifetime [14]. In our population, 28 patients (31.1%) had pulmonary hypertension, with no stratification by the type of CTD. The median time from diagnosis of ILD to the first hospitalization was 1.42 years in our study. In the study done by Brown et al. mean time from the diagnosis to respiratory admission was 6.3 months [10]. The longer time to the hospitalization in our study might partially be explained by the limited access to care, however different dataset characteristics and lack of stratification by hospitalization type in our group limit the comparison of the two studies.

Out of the total of 96 study participants, 73 patients (76%) required at least one hospitalization. The hospitalization rate in our cohort was significantly higher compared to a study from a tertiary scleroderma center (76% vs. 37%) [15]. In our study, we found that hospitalization was associated with a statistically significant (*p* = 0.02) increased risk of death. Similarly, Ratwani et al. [12] showed that patients with CTD-ILD who had hospitalization due to pulmonary causes had increased chances of death during hospitalization as well as decreased survival after discharge. These results reinforce the fact that hospitalization events can serve as an independent prognostic factor for morbidity and mortality in CTD-ILD patients. In our study, we observed no deaths in the non-hospitalized group. However, it is important to mention that older age can also increase all-cause mortality, and is a confounder that can potentially explain higher mortality in our cohort [16].

When we stratified hospitalization events by the cause, 51/73 (70%) patients had a hospitalization for a cardiopulmonary reason. They were significantly older than those admitted with non-cardiopulmonary cause. This finding is somewhat different from what was previously described in a cohort of patients with IPF by Brown et al. [10] In their study, patients who underwent hospitalization for respiratory causes were slightly younger than the patients who were hospitalized for non-respiratory causes. We used cardiopulmonary hospitalizations rather than respiratory hospitalization because ultimately ILD has a profound effect on the cardiovascular system, and advanced disease can lead to the development of heart failure which by itself could increase the frequency of hospitalization and overall survival. Also, it is possible that to some extent cardiopulmonary admissions in our patient population could be explained by the high cardiovascular comorbidity burden in the Bronx [17]. Additionally, our results showed that a length of stay greater than 7 days for cardiopulmonary cause (HR 4.82, *p* = 0.01) was associated with a higher risk of mortality similar to reported literature [15] (Fig. 2). Similar results were presented by Ratwani et al., where patients with prolonged hospitalization had worse overall outcomes [12].

One of the strengths of our study is that it collected patient-level data from a diverse cohort of Black and Hispanic patients from our community that are frequently underrepresented in other studies. We stratified the cause of hospitalization to help identify the patients at the highest risk. Further, our study spanned over 11 years, which is sufficient time to achieve the endpoint from the time of diagnosis [18].

There are some limitations to our study. First of all, observational study design makes the study more prone to biases and confounding which cannot be used to determine a causal relationship. We had to exclude a significant number of patients after our initial search due to a lack of adequate clinical data which resulted in a smaller sample size. It is possible that our study is underpowered, and that could have led to type II error when comparing variables.

## Conclusion

In conclusion, we demonstrated that hospitalizations for any cause, advanced age and male sex are associated with higher mortality. Hospitalization for cardiopulmonary causes, especially with a length of stay exceeding 7 days is a poor prognostic factor for patients with CTD-ILD and is associated with increased mortality risk. These findings can help to identify high-risk patients during hospital admission so that they can be monitored closely after discharge. Furthermore, additional efforts are needed to promote multi-disciplinary care models to identify and triage patients at high risk of complications related to CTD-ILD.

## Data Availability

The datasets used and analyzed during the current study are available from the corresponding author on reasonable request and in supplementary information files.
